# Limiting radial pedal forces greatly reduces maximal power output and efficiency in sprint cycling: an optimal control study

**DOI:** 10.1152/japplphysiol.00733.2021

**Published:** 2023-02-24

**Authors:** Dinant A. Kistemaker, Thijs M. Terwiel, Edwin D. H. M. Reuvers, Maarten F. Bobbert

**Affiliations:** Department of Human Movement Sciences, Vrije Universiteit Amsterdam, Amsterdam, The Netherlands

**Keywords:** cycling technique, effective force, energetics, index of effectiveness, optimal control

## Abstract

A cyclist’s performance depends critically on the generated average mechanical power output (AMPO). The instantaneous mechanical power output equals the product of crank angular velocity, crank length, and the tangential pedal force. Radial pedal forces do not contribute to mechanical power. It has been suggested that radial pedal forces arise from suboptimal pedaling technique and that limiting these would increase AMPO and efficiency. Here, we presented an optimal control musculoskeletal model of a cyclist (consisting of five segments driven by nine Hill-type muscle-tendon units) to predict maximal AMPO during sprint cycling at different levels of allowed radial pedal forces. Our findings showed that limiting radial pedal forces has a detrimental effect on maximal AMPO; it dropped from 1,115 W without a limit on radial forces to 528 W when no radial forces were allowed (both at 110 rpm). We explained that avoiding radial pedal forces causes ineffective use of muscles: muscles deliver less positive power and have a higher muscle power dissipation ratio (average mechanical power dissipated per unit of average positive power delivered). We concluded that radial pedal forces are an unavoidable by-product when optimizing for maximal AMPO and that limiting these leads to a performance decrease.

**NEW & NOTEWORTHY** In the literature, but also in the “cycling field” [e.g., trainers, coaches, and (professional) cyclists], it is often suggested that trying to limit/avoid radial pedal forces enhances cycling technique and with that maximal average power output and efficiency. In this paper, we introduce an optimal control model of a human cyclists (consisting of five segments and driven by nine Hill-type muscle-tendon complex models). With that we not only show, but also explain why limiting radial forces is a bad idea: it will decrease maximal attainable AMPO and will decrease efficiency.

## Graphical abstract

**Figure:**
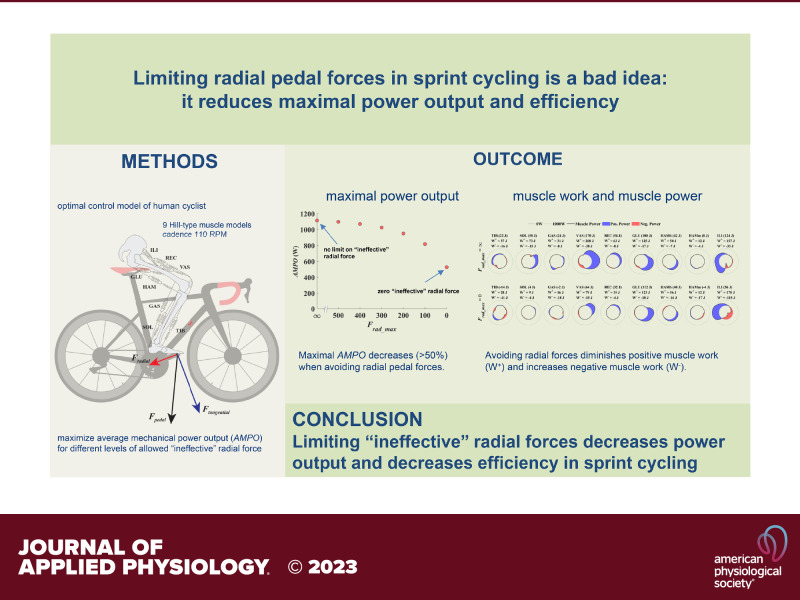

## INTRODUCTION

**Figure 1.**
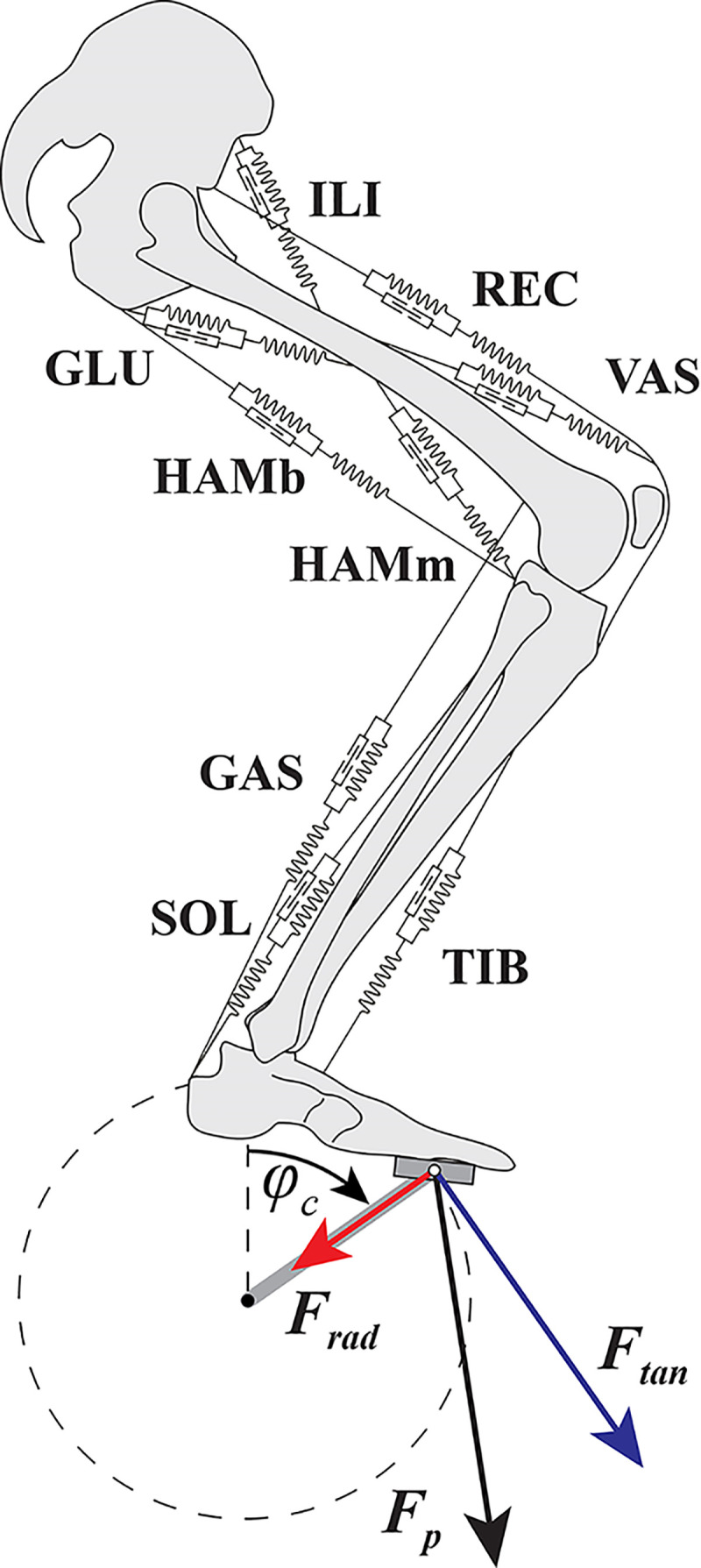
Schematic representation of the musculoskeletal model used to simulate cycling. The skeleton and crank were modeled as rigid segments connected in frictionless hinge joints and actuated by nine Hill-type muscle-tendon complexes. Pedal forces (*F*_p_; black arrow) are defined as the force from the pedal on the crank. Also shown are the radial (*F*_rad_; red arrow) and tangential (*F*_tan_; blue arrow) pedal force component. GAS, m. gastrocnemius; GLU, m. gluteus maximus; HAMb, bi-articular hamstring; HAMm, mono-articular hamstring; ILI, m. iliopsoas; REC, m. rectus femoris; SOL, m. soleus; TIB, m. tibialis anterior; VAS, mm. vastii.

The question has often been raised in the literature that pedaling technique in cycling leads to maximal performance (e.g., see Refs. 1–7). Performance of a cyclist depends critically on the average mechanical power output (AMPO) generated by the cyclist and the average mechanical power lost to the environment (8). To start with the obvious, mechanical power is produced by the force on the pedals. This pedal force does not influence the (typical) circular pedal path that is a consequence of the design of the bike (i.e., a stiff crank that revolves around the bottom bracket). Therefore, the cyclist has the freedom to choose the direction of the pedal force without influencing the pedal path. However, the direction of the pedal force does influence the crank power. The instantaneous mechanical power delivered to the crank by the cyclist equals the product of crank angular velocity and the moment produced by the cyclist on the crank. This moment, in turn, is the product of crank length and the so-called “effective force”; the component of the force from the cyclist’s pedal (*F*_p_) on the crank that is tangential to the crank motion (*F*_tan_, see Fig. 1). The radial component of the pedal force (*F*_rad_) is known as the “ineffective force” (e.g., see Refs. 1, 2, and 4) or “wasted force” (9), because it does not contribute to power output.

The fact that the radial component does not contribute to the mechanical power output has led Lafortune and Cavanagh (10) to introduce the index of effectiveness (IE): the magnitude of *F*_tan_ and the magnitude of *F*_p_ are each integrated over a complete crank cycle, and IE is calculated as the ratio of the two integrals, also known as the “motive efficiency” (11, 12) or “force effectiveness” (e.g., see Ref. 4). In all-out sprint cycling, the value of IE has a maximum of ∼0.8 at low pedaling rates and drops to values below 0.3 at high pedaling rates (13). In a 1-h endurance time trial on an ergometer, IE values range from ∼0.6 to ∼0.8 among top cyclists (14). From the finding that IE over a full cycle is less than 1, it follows that the pedal force is not purely tangential over the full crank cycle. In fact, it has been shown that substantial radial pedal forces occur, especially during the down stroke and around bottom-dead-center (13, 15). Various authors have taken the value IE to be an indicator of pedaling technique (3–5, 16–20) and thus implicitly assumed that the radial pedal forces during cycling arise from suboptimal pedaling technique. Based on this assumption, it has been proposed that improving IE is beneficial for mechanical power output (1) and gross efficiency in endurance cycling (4, 20, 21). In fact, commercially available power meters (e.g., Pioneer Pedaling Monitor System) have the option to provide riders with IE and supporting material states that cyclists should train such to increase IE to arrive at a better cycling technique/higher cycling efficiency.

Why would a cyclist produce metabolically costly muscle forces to generate radial pedal forces that do not contribute to mechanical power output? Is human (elite) cycling indeed suboptimal, or are radial pedal forces an unavoidable by-product when maximizing AMPO? In the first case, avoiding radial pedal forces may increase maximal attainable AMPO. In the second case, it is expected that limiting or avoiding radial pedal forces will decrease maximal attainable AMPO. In general, if a cyclist’s goal is to optimize the controls (i.e., muscle activations over time) to deliver maximal AMPO during a full cycle, first principles of optimal control theory tells us that the addition of a constraint on the controls (i.e., to avoid radial pedal forces) can never lead to an increase in AMPO. Furthermore, several studies indeed indicate that limiting radial pedal forces is not a good idea. For example, Kautz and Hull (22) decomposed pedal forces in cycling and showed that radial pedal forces were partly caused by outwardly directed inertial forces, which increased with cycle frequency, and by gravity. The authors argued that eliminating nonmuscular radial force contributions would require muscles to produce opposite radial pedal forces, which is metabolically costly. In line with this argument, Höchtl et al. (23) showed that radial pedal forces also occur if muscle activation in a forward simulation model of cycling is optimized for minimal metabolic energy consumption at a given AMPO, causing them to conclude that a certain amount of radial pedal force is needed for a pedaling technique that is optimal for efficiency.

Although it is true that inertial forces and gravity contribute to radial pedal forces, there is also a (much more substantial) muscular contribution to radial pedal forces (22). Onasch and Herzog (24) studied isometric situations on a bicycle and as such cancelled these inertial forces. They showed for different crank angles that the maximal isometric “effective” tangential pedal force was smaller when subjects were required to direct the pedal force perpendicular to the crank than when they were free to choose the force direction. Their explanation was as follows. Orienting the force on the pedal in a specific direction requires a specific muscle synergy. If the task is to produce a maximal tangential pedal force without constraint on the direction of the pedal force, subjects are free to choose the optimal synergy that yields the largest tangential force; in that case the corresponding direction of the pedal force is an irrelevant outcome (25). Yet, if the direction of the pedal force to be produced is constrained, subjects are forced to choose a synergy different from the optimal one; the corresponding maximal tangential force component of that synergy will be smaller than in the unconstrained condition. This result indicates that the radial pedal forces in cycling may occur as a “by-product” for maximal tangential pedal force. An analysis of cycling as done by Onasch and Herzog (24) provides valuable insight in the maximal static tangential pedal force, but does not necessarily translate to actual sprint cycling: in sprint cycling the task is to maximize the AMPO and this is not the same as maximizing the tangential pedal force at each pedal position (see discussion). Furthermore, a static analysis by definition cannot answer the question why limiting or avoiding radial pedal forces would lead to a reduced maximal attainable AMPO and by how much it would be reduced.

In this study, we present an optimal control musculoskeletal model of a human cyclist to predict maximal AMPO during sprint cycling at different levels of allowed radial pedal forces. In previous simulation studies on sprint cycling, we used so-called “bang-bang” control (meaning that muscles were either switched fully on or off), which resulted in a straightforward optimization problem that can be readily tackled with conventional shooting optimization techniques (26–28). In the present study, we will impose constraints to limit radial pedal forces, which greatly increase the complexity of the optimization problem (e.g., “bang-bang” control is no longer optimal). To allow for continuous change in muscle activation, we applied techniques from the optimal control framework (e.g., direct collocation and a sparse nonlinear optimizer; see Refs. 29–31, 47). For the interested reader on applying the optimal control framework to musculoskeletal models, we refer to the excellent work of the group of van den Bogert (32–34) and of De Groote (35, 36). Using our optimal control model of a human cyclist, we will show and explain that constraining pedal forces to avoid radial pedal forces—although leading to a higher IE—will lead to a substantially lowered maximal attainable AMPO and increased negative muscle power in sprint cycling.

## METHODS

### Musculoskeletal Model

We used a two-dimensional musculoskeletal model (see Fig. 1) similar to models used in studying vertical jumping (e.g., see Refs. 37 and 38) and sprint cycling (26, 28). In short, the skeletal model consisted of five rigid segments connected in frictionless hinge joints. These segments represented the crank (0.17 m), foot (from ankle to crank: 0.165 m), lower leg (0.458 m), upper leg (0.485 m), and the “head-arms-trunk” (HAT; 0.82 m). The position of the hip and angle of HAT were fixed in space. The crank angle (φ_cr_) was defined to be zero when the crank was upright (top-dead-center) and crank angular velocity was defined positive when rotating clockwise (see Fig. 1). The crank’s origin was fixed in space and the crank angular velocity was fixed to have a constant value. Setting the crank velocity to a fixed value results in isokinetic cycling and is mechanically similar to having an infinite “effective inertia” (i.e., the inertia “felt” on the crank that depends on (rotational) inertia of the bike components and gear ratio; see Ref. 39). Since maximal AMPO is typically delivered at high speeds and thus involving high gear ratios, effective inertia of a sprint cyclist is very high and hence cycling is well approximated by a fixed angular velocity (see Ref. 28). The advantage of having a fixed crank angular velocity is that it greatly simplifies the optimization problem, as the two legs are mechanically decoupled (the torques delivered by one leg do not influence the accelerations of the other) and there is no need to model all the resistive and inertial forces acting on the cyclists (see Ref. 39). As such, only the muscle activations of one leg need to be optimized; the total AMPO is then simply twice that of one leg. Equations of motion for the skeletal model plus crank were derived using a Newton–Euler approach as described in Ref. 40. The position of the hip relative to the crank’s origin was optimized for maximal AMPO (see later).

The model was driven by nine Hill-type muscle tendon units representing m. iliopsoas (ILI), m. gluteus maximus (GLU), mm. vastii (m. vastus medialis + m. vastus lateralis + m. vastus intermedius; VAS), m. rectus femoris (REC), the bi-articular hamstring (m. biceps femoris caput longum, m. semimembranosus, m. semitendinosus; HAMb), the mono-articular hamstring (m. biceps femoris caput breve), m. gastrocnemius (GAS), m. soleus (SOL), and m. tibialis anterior (TIB). Every muscle tendon complex consisted of a contractile element (CE) and a series elastic element (SE). In contrast to previous work, we removed the parallel elastic element (PE) as we found that it was not delivering force in the optimal cycling motions but did slow down convergence to the optimal solutions. Activation dynamics, describing the relation between neural input to the muscle (STIM) and active state (*q*), here defined as the relative amount of Ca^2+^ bound to troponinC (41), was modeled according to Hatze (42, see also Ref. 43). This model of activation dynamics first describes the dynamical relationship between the relative amount of intracellular Ca^2+^ (γ_rel_) and STIM, the normalized α-motoneuron firing frequency. The model then nonlinearly relates active state (*q*) to γ and the length on the CE (*l*_CE_). This last step accounts for the *l*_CE_ dependent [Ca^2+^] sensitivity of muscle fibers (see Ref. 43). Contraction dynamics describes how the muscle delivers force depending on *q*, *l*_CE_, and CE contraction velocity (*v*_CE_) and was modeled in two steps. First, we used a parabola to describe the maximal isometric force-length relationship of CE. This force is multiplied with *q*, and thus together with the activation dynamics describes the muscle activation-dependent isometric force. Second, CE force was nonlinearly dependent on *v*_CE_. The eccentric force was modeled using a hyperbola. The concentric part was described using a classical Hill curve for which the maximal shortening speed was made dependent on *q* and *l*_CE_. For a detailed overview of all modeled steps and all nonmuscle-specific parameters, please refer to our previous papers (e.g., see Refs. 37, 44, and 45).

All muscle-specific parameters were the same as used in our previous studies on sprint cycling (see e.g., Refs. 26 and 28), apart from the hamstrings that were split in a mono- and bi-articular part (parameters based on Ref. 46) and are shown in Table 1. To make the muscle model suitable for a direct collocation approach (see later), the force-length-velocity relationship and activation dynamics were slightly reformulated to a continuous and differentiable form (see also Ref. 47). The musculoskeletal model as a whole had 26 states (9 *l*_CE_, 9 γ_rel_, 4 segment angles, and 4 segment angular velocities). The trunk was implicitly modeled with a fixed angle and zero angular velocity. The independent inputs to the model were the crank angular velocity (set to a fixed value) and STIM.

**Table 1.** Muscle-specific parameters

Muscle	*F*_max_, N	*l*_CE_Opt_, m	*l*_SE_0_, m	*a*_0_, m	*a*_1A_, m	*a*_1K_, m	*a*_1H,_ m	*a*_2A_, m	*a*_2K_, m
TIB	1,200	0.087	0.317	0.448	0.037	0	0	0	0
SOL	3,000	0.055	0.246	0.23	−0.0627	0	0	−0.0081	0
GAS	1,500	0.055	0.382	0.394	−0.0627	−0.0186	0	−0.0081	0.0015
VAS	5,250	0.093	0.160	0.231	0	0.042	0	0	0
REC	1,750	0.081	0.340	0.418	0	0.042	0.035	0	0
GLU	2,750	0.200	0.150	0.255	0	0	−0.062	0	0
HAMb	1,925	0.104	0.370	0.41	0	−0.026	−0.077	0	0
HAMm	275	0.110	0.200	0.339	0	−0.026	0	0	0
ILI	4,000	0.102	0.115	0.248	0	0	0.05	0	0

Muscle-specific parameters. *F*_max_ = maximal isometric contractile element (CE) force, *l*_CE_opt_ = optimum CE length and *l*_SE_0_ = series elastic element slack length. Parameters *a* define the muscle-tendon complex length (*l*_MTC_) as a function of the ankle (φ_A_), knee (φ_K_), and hip (φ_H_) joint angle. The muscle-tendon complex length for each muscle as a function of the joint angles is given by a sum of second order polynomials with the polynomial constants provided in Table 1. For example, *l*_MTC_HAMb_ = *a*_0_ + *a*_1A_*·*φ_A_ + *a*_2A_*·*φ_A_^2^ + *a*_1K_*·*φ_K_ + *a*_2_*_K_·*φ_K_^2^ + *a*_1H_*·*φ_H_ + *a*_2H_*·*φ_H_^2^. Since *a*_1A_, *a*_2A_, and *a*_2K_ are zero for this muscle, and that *a*_2H_ is zero for all muscles (and not given in Table 1), this simplifies to: *l*_MTC_HAMb_ = *a*_0_ + *a*_1_*_K_ ·* φ_K_ + *a*_1H_ *·* φ_H_. The moment arm (arm) of a particular muscle around a particular joint is found by taking the partial derivative of *l*_MTC_ with respect to that joint. For example, arm_HAMb_*___*= *a*_1_*_K_* (note that since *a_2_*_K_ = 0, the moment arm of the bi-articular hamstring (HAMb) around the knee is constant, and not dependent on the knee joint angle [this in contrast to, for example, the moment arm of m. gastrocnemius (GAS)]. TIB, m. tibialis anterior; SOL, m. soleus; GAS, m. gastrocnemius; VAS, mm. Vastii; REC, m. rectus femoris; GLU, m. gluteus maximus; HAMb, bi-articular hamstrings; HAMm, mono-articular hamstrings; ILI, m. Iliopsoas.

### Constrained Optimization

The goal of the optimization was to find the optimal muscle activations over time [STIM(*t*)] that maximized the average mechanical power output during a full cycle (AMPO), for different levels of allowed radial pedal forces. The optimization criterion for our (constrained) optimization problem was to maximize the total amount of mechanical work done on the crank (*W*_cr_) over a full crank cycle with a particular cycle frequency (cf):

Wcr=∫01cfMcr · φ˙cr dt=∫01cfFtan·lcr· φ˙cr dt.

Here, *M*_cr_ represents the torque around the bottom bracket on the crank, ${\dot{\varphi }}_{\text{cr}}=2\pi \cdot \text{cf}$ represents the crank angular velocity and is positive when the crank rotates clockwise (see Fig. 1). Alternatively, *W*_cr_ can be expressed in terms of *F*_tan_ and the length of the crank (*l*_cr_). AMPO equals *W*_cr_·cf.

STIM(*t*) for the nine modeled muscles of one leg were optimized to maximize *W*_cr_. We only needed to optimize STIM(*t*) for one leg as during isokinetic cycling legs are mechanically decoupled and we assumed symmetry between the legs. In the remainder of this article, we will provide values of the left and right leg muscles together, unless specifically stated otherwise. To ensure fully periodic behavior, we added constraints on all 9 STIM(*t*) and 26 states of the musculoskeletal model [x(*t*)] such that [STIM(0) x(0)]=[STIM(1/cf) x(1/cf)]. Finally, to investigate the influence of IE on maximal AMPO, we defined seven conditions by constraining the instantaneous radial pedal force at different values:

$$\left|F_{\text{rad}}(t)\right|\leq F_{\text{rad\_max}},$$

with $\left|F_{\text{rad}}(t)\right|$ the absolute of the radial pedal force and *F*_rad_max_ set to either 0, 100, 200, 300, 400, 500, or ∞ N. When F_rad_max_ was set to 0 N, no radial pedal forces were allowed; hence IE is one. When, for example, *F*_rad_max_ was set to 200 N, maximal allowed instantaneous radial pedal force was 200 N, and *F*_rad_max_ when set to *∞*N there was no limit on *F*_rad_.

To identify optimal STIM(*t*), we used a direct collocation approach (48). To do so, all states and inputs were parameterized and all differential equations of the musculoskeletal model (activation, contraction, and skeletal dynamics) were discretized at every collocation point (30, 34, 35, 47). These discretized differential equations, together with the constraint equations and optimization criterion described earlier, form a large-scale nonlinear programming problem, which was solved using sparse nonlinear optimal controller (SNOPT; TOMLAB Optimization, Pullman, WA). The derivatives and second derivatives of the constraints and cost function were computed analytically using PROPT (TOMLAB Optimization, Pullman, WA).

To check if we used enough collocation points to adequately discretize the musculoskeletal dynamics, we performed a forward simulation with the results obtained from the optimal control model. It was found that the states obtained from a forward simulation and those obtained from our optimal control model were nearly identical when using 50 collocation points (on average ∼1 collocation point per 10 ms).

To avoid the possibilities of arriving at (getting stuck in) a local minimum, each condition was optimized ten times, with each optimization starting from a random initial guess for all parameterized inputs and states. For each condition, we selected the solution leading to the highest AMPO.

### Optimal Cycling Frequency and Saddle Position

Before investigating the influence of limiting radial pedal forces, we first identified the optimal cf and saddle position for unconstrained cycling (with *F*_rad_max_ = ∞) To do so, we added three additional optimization parameters to the optimal control problem described earlier, being cf itself and the *x*- and *y*-position of the hip with respect to the position of the bottom bracket. We then found the optimal cf and the hip position simultaneously with STIM(*t*) that maximized AMPO. For all other optimizations, we fixed cf and hip position to these values.

### Index of Effectiveness

In the literature, there seems to be a lack of consensus over the exact definition of IE; the index of effectiveness (cf. Refs. 1, 5, 6, 10, 14, and 20). Moreover, sometimes IE is defined unclearly or even in a mathematically unsound way. Although different definitions (e.g., using *F*_tan_ or the absolute of *F*_tan_) may lead to (slightly) different values, we found that for this study these differences only marginally affected our solutions. Here, we have chosen to define IE along the lines of Lafortune and Cavanagh (10): IE is the ratio of the magnitude of *F*_tan_ and the magnitude of *F*_p_ both integrated over crank angle ($\varphi_{\text{cr}}$) for a complete cycle:

$$\text{IE}=\frac{\int_{0}^{2\pi }\left|F_{\tan}\right|{\text{dφ}}_{\text{cr}}}{\int_{0}^{2\pi }\left|F_{\text{p}}\right|{\text{dφ}}_{\text{cr}}},$$

*F*_tan_ was defined positive when it pointed in the direction of pedal movement and *F*_rad_ was defined positive when it pointed (inward) to the crank origin (see also Fig. 1).

### PDR; Average Muscle Mechanical Power Dissipation Ratio

To assess and compare the contribution of all muscles to the average positive and negative mechanical power delivered at different values of AMPO, we define average mechanical muscle power dissipation ratio (PDR); the ratio of average negative mechanical power dissipated (AMPO^−^) per unit of average positive power delivered (AMPO^+^) in one full cycle:

PDR=AMPO−AMPO+=cf·∑n=19∫01cfPMTC_n −dtcf·∑n=19∫01cfPMTC_n+ dt=cf·∑n=19∫01cf[FMTC_n·vMTC_n]−dtcf·∑n=19∫01cf[FMTC_n·vMTC_n]+dt,

*P*_MTC_*__n_* is the instantaneous mechanical power delivered by the *n*th muscle-tendon complex and equals the product of the muscle-tendon complex force (*F*_MTC_*__n_*) and contraction velocity (*v*_MTC_*__n_*). $P_{\text{MTC}\_n}^{-}$ denotes the negative instantaneous mechanical power of muscle *n*; it equaled $P_{\text{MTC}\_n}$ when it was ≤0 and was set to 0 otherwise. Likewise, $P_{\text{MTC}\_n}^{+}$ denotes the positive instantaneous mechanical power of muscle *n*.

## RESULTS

### Optimal Saddle Position and Cycle Frequency

We first identified the optimal cf and hip position that yielded the highest AMPO. The identified optimal pedaling rate for unconstrained cycling was very close to 110 rpm. The results in terms of kinematics and AMPO were close to that found in Ref. 28, and close to the pedaling rate observed experimentally in sprint cycling (49). For this article, cf was fixed to 1.83 Hz (equaling to cycle frequency of 110 rpm). The hip position was set to the optimal hip position for unconstrained cycling, which was [−0.055 0.87] m (effective seat tube angle of 1.63 rad with respect to positive *x*-axis) relative to the origin of the crank (bottom bracket) and was close to that used by van Soest and Casius (27).

### Convergence of Optimal Control Solutions

We were able to successfully identify the optimal STIM(*t*) for maximal AMPO for all levels of constrained radial forces investigated. Furthermore, we were successful in constraining radial pedal forces at different levels of maximally allowed radial pedal forces. For example, the maximal value of radial force in the condition in which no radial pedal forces were allowed (*F*_rad_max_ = 0) was only ∼2 ×·10^−9^ N (rms = ∼5 ×·10^−10^ N). Visualizations of the cycling model for *F*_rad_max_ = ∞ and *F*_rad_max_ = 0 can be found online (Supplemental Material; see https://doi.org/10.6084/m9.figshare.21711020).

### Comparison with Literature

The unconstrained (*F*_rad_max_ = *∞*) AMPO was found to be 1,115 W, which agrees with values reported in the literature on sprint cycling (see Table 2). In addition, the identified maximal AMPO is very close to that found by van Soest and Casius (28). Note that this is not trivial, since they used a different approach in which they used “bang-bang” control (muscles were allowed to switched fully on or off once during each crank cycle) and in which they added the constraints on periodicity (see methods) as penalty terms in their optimization criterion. Reassuringly, in our optimal control solution for the unconstrained condition, we found that the muscle activation pattern was similar to earlier work of our group: muscles were either fully turned on or turned off (see also Ref. 27).

**Table 2.** Reported values for two-leg AMPO in the literature on sprint cycling

Source	AMPO
Dorel et al. (2010) (13)	1,250 ± 117 (*n* = 14) W
Vandewalle et al. (1987) (49)^a^	1,150 ± 127 (*n* = 7) W
Elmer et al. (2011) (50)^b^	1,126 ± 38 (*n* = 11) W
McDaniel et al. (2014) (51)^b^	1,098 ± 34 (*n* = 10) W
Martin and Brown (2009) (52)^b^	1,080 ± 62 (*n* = 13) W
Van Soest and Casius (2000) (28)	1,076 W
Current study	1,115 W

AMPO, average mechanical power output.

a Vandewalle et al. reported on many different types of athletes; the value displayed here is for track cyclists (the other types of athletes scored similar values).

b These studies only reported power output for one leg; right-left symmetry has been assumed to calculate the AMPO for two legs.

**Figure 2.**
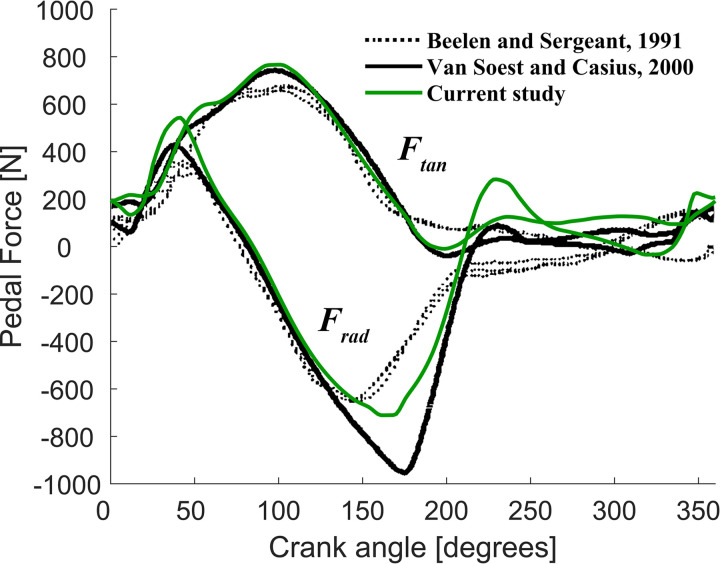
Tangential (*F*_tan_) and radial (*F*_rad_) component of the pedal force as a function of crank angle. Crank angle is equal to that defined in Fig. 2 (0° is top-dead center). Dotted lines are the experimentally observed force histories of three full crank cycles of a typical subject cycling at 120 rpm (15). Solid black lines are the pedal force histories obtained by Van Soest and Casius (28) and the green solid lines are the pedal force histories of our optimal control model cycling at 110 rpm.

Figure 2 shows the optimal unconstrained (*F*_rad_max_ = ∞) radial and tangential pedal forces as a function of crank angle found in this study, as well as those found by van Soest and Casius (28) and those obtained experimentally by Beelen and Sargeant (15). In general, the tangential pedal forces profiles look very similar. As with the study of van Soest and Casius (28), the radial pedal forces of the model are substantially higher around bottom-dead-center (crank angle = 180°) than experimental values. These higher radial pedal forces occur when tangential pedal forces are very small and thus when little power is delivered. As argued by van Soest and Casius (28), it may be that subjects avoid high radial pedal forces when mechanical power output is low. Be that as it may, we found that the radial pedal forces at bottom-dead-center were also influenced by the positioning of the hip, as illustrated by the differences in peak radial force between this study (note that we optimized the hip position for maximal AMPO for *F*_rad_max_ = ∞; see methods) and that of van Soest and Casius (28). In addition, we found that peak radial pedal forces were influenced by the values of the maximal isometric forces of the muscles modeled (data not shown). Since the values of the muscle properties originate from elite jumpers (37, 53), it is very well possible that the differences in radial pedal forces between our model and those of elite cyclists are partly caused by differences in muscle properties (see also Ref. 27). Nevertheless, we believe that our model is capable of adequately capturing the important features of real cycling (visualizations of the model can be found in Supplemental Data).

### Influence of Limiting Radial Pedal Forces on Maximal Attainable AMPO

**Figure 3.**
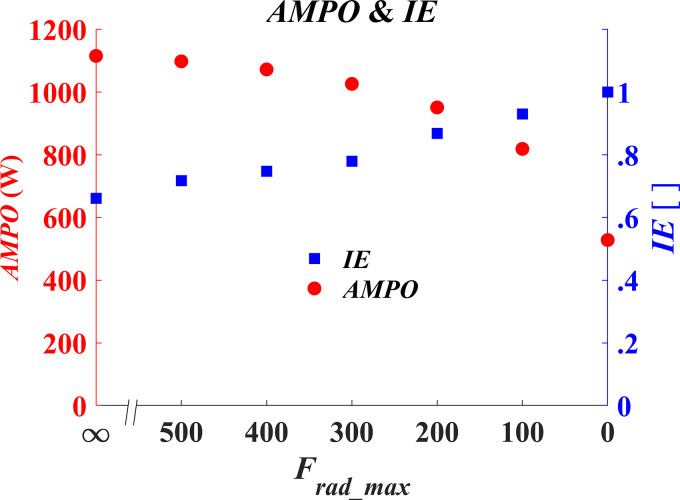
Maximal attainable average mechanical power output (AMPO) and accompanying index of effectiveness (IE) as a function of the maximal allowed “ineffective” radial pedal force.

In Fig. 3, AMPO is plotted as a function of maximally allowed radial pedal force. As expected (see introduction), the highest value of AMPO was found when the radial force was unconstrained (*F*_rad_max_ = ∞): 1,115 W. Also in line with our expectations is the clear reduction in AMPO with a reduction in the allowed radial force. When no radial pedal forces were allowed, maximal AMPO was reduced by as much as 53%, to a value of 528 W. In Fig. 3, we also plotted the IE calculated from our optimal solutions; clearly the highest IE does not coincide with the highest attainable AMPO. This furthermore indicates that the radial pedal forces are, as suggested in introduction, unavoidable “by-products” when maximizing AMPO.

### Influence of Limiting Radial Pedal Forces on Maximal Tangential Pedal Forces

**Figure 4.**
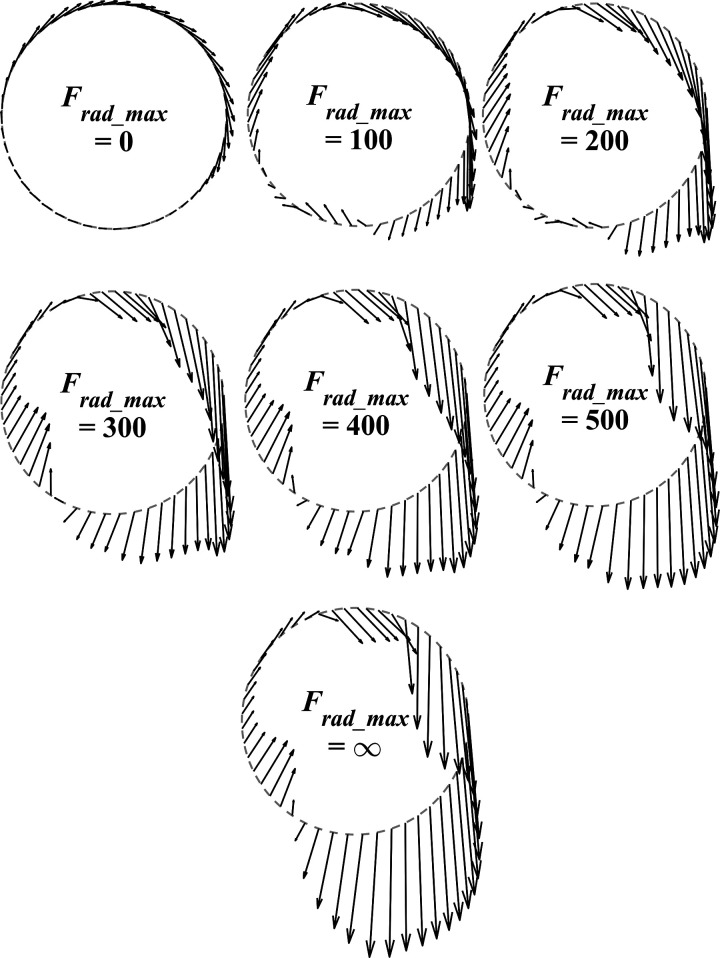
Pedal force as a function of crank angle, for all seven conditions. Pedal forces were drawn as vectors at fixed crank angle intervals.

**Figure 5.**
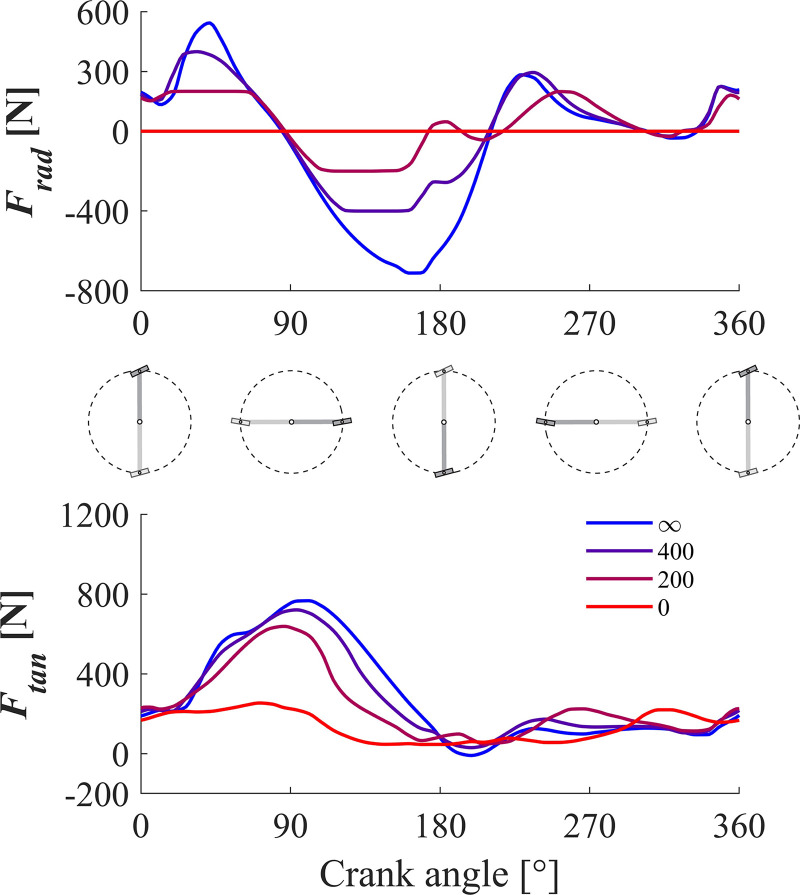
Tangential (*F*_tan_) and radial (*F*_rad_) component of the pedal force as a function of crank angle for four different conditions.

To understand why reducing the maximal allowed radial pedal force reduces the maximal attainable AMPO, we first looked at the pedal forces. In Fig. 4, the optimal pedal force as a function of crank angle is plotted at fixed intervals for all levels of *F*_tan_max_ investigated. Clearly, the magnitude of the total pedal force (length of the arrows) increased monotonically with increased allowed radial pedal forces. This is even more apparent when plotting time histories of *F*_rad_ and *F*_tan_ as a function of crank angle for different levels of *F*_rad_max_ (Fig. 5). When no radial pedal forces were allowed, pedal forces were obviously always tangential (i.e., *F*_rad_ = 0), but apparently this constraint comes with a substantial cost: tangential pedal forces are much lower, leading to a reduced AMPO. This leads to the obvious question: why is AMPO reduced when limiting radial pedal forces? Here, we will identify the muscles responsible for loss in power of our model, and in discussion we will try to unravel the underlying mechanism.

### Influence of Limiting Radial Pedal Forces on Muscle Power

**Figure 6.**
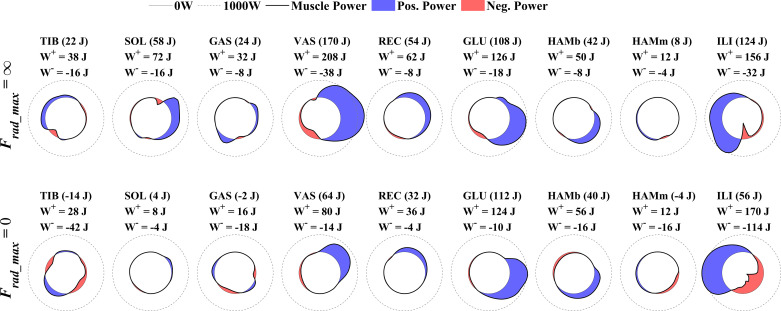
Instantaneous mechanical (muscle tendon complex) power delivered by each of the modeled muscles as a function of crank angle. *Top row*: unconstrained (*F*_rad_max_ = ∞) cycling. *Bottom row*: fully constrained (*F*_rad_max_ = 0). Outer dashed circle was drawn at 1,000 W and the inner solid line circle at 0 W. Blue denotes when muscles deliver positive power and red when delivering negative power. The total work, total positive work (*W*^+^) and total negative work (*W*^−^) done during one full cycle are given for each muscle separately. Note that the blue and red area scales (nonlinearly) with positive and negative work. Furthermore, note that average mechanical power output (AMPO) equals the sum of all work done by each muscle multiplied by the cycle frequency (=1.83 Hz).

In Fig. 6, we show for each modeled muscle (summed over both legs) the net mechanical work, total positive work (*W*^+^) and the total negative work (*W*^−^) done during one full crank cycle (note that AMPO equals net mechanical work delivered during one crank revolution multiplied by cycle frequency). In addition, we show for each muscle (summed over both legs) the instantaneous power delivered as a function of crank angle during one full crank revolution. This is shown for both the unconstrained (*F*_rad_max_ = ∞) and the fully constrained situation (*F*_rad_max_ = 0). Three clear observations about loss in power production can be made when closely observing muscle power. First, the biggest difference in average power production is caused by a reduction in power of mm. vasti: these muscles deliver ∼195 W less per cycle when avoiding radial pedal forces. Second, the muscles spanning the ankle joint are not capable of delivering power when avoiding radial pedal forces. In fact, instead of delivering positive power at a rate of 185 W, the muscles spanning the ankle joint deliver −21 W in the fully constrained condition; note that delivering negative power means that that they dissipated power. The third main reason for the loss in AMPO in the maximally constrained situation is a reduction of net power of m. iliopsoas. Even though m. iliopsoas delivers, perhaps counter-intuitively, more positive power when radial pedal forces are limited (by 25 W), it also delivers much more negative power (by −150 W); hence the contribution of m. iliopsoas to AMPO drops by ∼125 W. In summary, constraining radial pedal forces causes mm. vastii to deliver much less positive power, causes plantar flexors to stop producing any power, and causes m. iliopsoas to dissipate more power. From the individual muscle contributions to AMPO, we calculated the average muscle mechanical power dissipation ratio (see methods). For the unconstrained condition, PDR was −0.20 and increased to −0.45 in the fully constrained condition.

## DISCUSSION

In this study, we presented an optimal control model of a cyclist and showed that radial pedal forces in cycling occur as a “by-product” for maximal tangential pedal force, which means that reducing radial pedal forces reduces the maximal average mechanical power output (AMPO). When completely avoiding radial pedal forces (leading to a “perfect” IE of 1), maximal AMPO during one cycle drops by more than 50% compared with the unconstrained condition (leading to an IE of 0.66). In the next section, we will first attempt to explain the mechanisms responsible for the decrease in AMPO when avoiding radial pedal forces. Then we will argue that sprint cycling efficiency will also decrease when avoiding radial pedal forces.

### Why Does AMPO Decrease When Avoiding Radial Pedal Forces?

**Figure 7.**
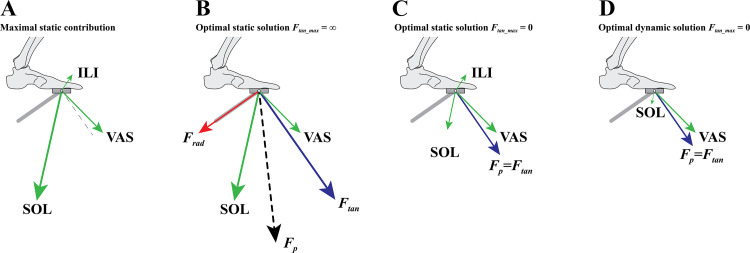
Illustration of the maximal static *F*_tan_ from three muscles (note that the length of the vectors are not drawn to size; see appendix). *A*: maximal pedal force contribution of three muscles (green arrows). Note that the contribution of a muscle to the pedal force diminishes with increasing distance from the pedal to the joint(s) that muscle spans (see also appendix). *B*: optimal muscle force distribution for maximal *F*_tan_ (blue arrow) when *F*_rad_ is unconstrained (*F*_rad_max_ = ∞). mm. vastii (VAS) and m. soleus (SOL) can be activated maximally. The pedal force (*F*_p_; black dashed arrow) has a substantial radial component (*F*_rad_; red arrow) *C*: optimal muscle force distribution for maximal *F*_tan_ when *F*_rad_ is fully constrained (*F*_rad_max_ = 0). VAS is maximally activated. m. iliopsoas (ILI) cannot contribute to a positive *F*_tan_ but is maximally activated to compensate for submaximally activated SOL. Here, *F*_p_ equals *F*_tan_ since *F*_rad_ is constrained to be zero. *D*: optimal “quasi-static” solution; when taking into account capability of delivering positive instantaneous muscle power, m iliopsoas is no longer activated, which further reduces the activation of the m. soleus.

In Fig. 6, we showed the power delivered and work done by the individual muscles during a full cycle, both for unconstrained cycling and constrained cycling when completely avoiding radial pedal forces. It is clear that in the latter case, muscles not only deliver less positive power, but also deliver more negative power (and thus dissipate energy). To get an intuitive idea of why this is the case, we will look at the at the optimal isometric (static) contributions of three muscles (mm. vastii, m. iliopsoas, and m. soleus) to the pedal force (green arrows; Fig. 7) for a crank angle of ∼55° (see appendix for the calculation of the force contributions). As explained in appendix, in general, the contribution of a unit of force of a muscle to the pedal force decreases with increasing distance from joint(s) that the muscle spans to the pedal. Thus, during cycling, 1 N delivered with an ankle plantar flexor leads to a much higher pedal force than 1 N delivered by a hip flexor (in fact, up to two orders of magnitude). Without a constraint on radial pedal forces (*F*_rad_max_ = ∞), finding the optimal combination of isometric muscle forces for maximal *F*_tan_ is straightforward: just activate maximally all muscles that have a positive *F*_tan_ component; in this case both m. soleus as well as mm. vastii (Fig. 7*B*). This leads to a large *F*_tan_ (blue arrow) and a substantial *F*_rad_ (red arrow). When avoiding radial pedal forces, the optimal solution for maximal static *F*_tan_ is far less straightforward (Fig. 7*C*). The mm. vastii are activated maximally and the outward radial component of the generated pedal force is compensated by an inward radial component of m. soleus. However, since m. soleus has a much larger inward radial component than the outward *F*_rad_ component of mm. vastii [this is augmented as the ankle joint (spanned by the m. soleus) is closer to the pedal than the knee joint (spanned by the mm. vastii); see appendix], the activation of m. soleus is very limited. The activation of m. soleus can be increased if m. iliopsoas is activated, such that m. iliopsoas compensates for the inward *F*_rad_ component of m. soleus. This is beneficial as the positive *F*_tan_ component of the m. soleus is larger than the negative *F*_tan_ component of the m. iliopsoas. In the optimal isometric solution (*F*_tan_max_ = 0), m. soleus is submaximally activated, whereas m. iliopsoas—which negatively contributes *F*_tan_—needs to be fully activated to avoid radial pedal forces. Consistent with the experimental findings of Onasch and Herzog (24), our isometric muscle force optimization example shows that avoiding radial pedal forces leads to a decrease in activity of muscles that can contribute positively to *F*_tan_. In addition, it shows that avoiding radial pedal forces may lead to an increase in activity of muscles that contribute negatively to *F*_tan_.

Although a static/isometric analysis yields insight, it does not tell the full story. Several important factors play a role that complicate understanding the optimal muscle activation for maximal AMPO. First, activation dynamics causes a delay between the change in muscle activation and corresponding change in muscle force. In previous research, we have shown that activation dynamics plays an important role in explaining the optimal pedaling frequency (28) and the linear relationship between pedal force and crank angular velocity (27). Here, we show that activation dynamics augments the reduction in muscle power when avoiding radial pedal forces. For example, this is the explanation of the vast reduction in power (by 195 W) of mm. vastii. From about a crank angle of 135° to a crank angle of 180°, mm. vastii and m. gluteus maximus produce a large outward radial force. This radial force becomes very large mostly due to the mm. vastii (a muscle crossing the knee joint has a greater effect on the pedal force than a muscle crossing the hip joint; see appendix). Only the bi-articular hamstring can compensate for this outward radial force while producing positive power, but is much smaller than the mm. vastii. To avoid radial forces, the mm. vastii is switched off much earlier even though it can contribute greatly to power production. Due to activation dynamics, shutting this muscle group off needs to be done ahead in time. The result is that not only the mm. vastii are generating less instantaneous power, they are also doing so over a much smaller range in crank angles (cf. Fig. 6 VAS 1st and 2nd row). Since the m. gluteus maximus is crossing the hip joint, its effect on the radial pedal force is much smaller compared with that of the mm. vastii. The bi-articular hamstring can be fully activated to compensate for most of the outward radial forces caused by the m. gluteus maximus. In fact, the bi-articular hamstring is the only muscle that is not affected by the constraint on the pedal force (it actually delivers a little bit more power when *F*_rad_ = 0, but this is due to small differences in cycling kinematics). Around 135°, the inward radial force of the bi-articular hamstring is too small. The m. gluteus maximus needs to be shut off, but, due to activation dynamics this is relatively slow (and thus would cause a great decrease in positive power) and the optimal solution is to both decrease the activity of the m. gluteus maximus and to increase its antagonist the m. iliopsoas, causing a large increase in negative AMPO (from ∼−60 to −210 W cf. Fig. 6 ILI 1st and 2nd row).

**Figure 8.**
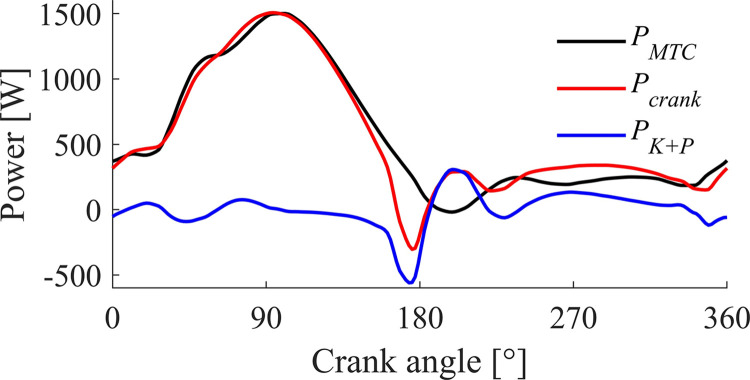
Time histories of the instantaneous mechanical power terms of our bike model as a function of crank angle for the optimal unconstraint solution. *P*_MTC_ = muscle-tendon complex power, *P*crank = mechanical power delivered on the crank, and *P*_K+P_ = sum of the rate of change of kinetic and potential energy. Given the definitions of directions of forces, the power equation equals ∑*P* = −*P*_MTC_ + *P*_crank_ = *P*_K+P_ (see Ref. 8).

A second complicating factor is that there is no direct relation between instantaneous crank power and instantaneous muscle power. As discussed earlier, in Fig. 7, we plotted the contribution of the muscle forces to the pedal forces. When looking at the power delivered by the muscles, such a static analysis is not insightful. It is true that over a full cycle average mechanical crank power equals average mechanical muscle power, because the movement is periodic. However, in general, the instantaneous crank power (*P*_crank_) is not equal to instantaneous muscle (−tendon complex) power (*P*_MTC_). *P*_MTC_ equals *P*_crank_ plus the rate of change in kinetic and potential energy [*P*_K_*_+_*_P_; 8, 54]. Figure 8 shows these power terms as a function of crank angle for the optimal unconstraint solution. We found that the peak rate of change in kinetic energy can be as large as −550 W (independent of a constraint on radial forces). Thus, maximizing instantaneous crank power would yield instantaneous joint accelerations that are inconsistent with a cycling motion.

A third complicating factor is that although muscle force contributions to the pedal force are insightful for understanding how to coordinate muscles to avoid radial pedal forces, they are not insightful when it comes to understanding how (instantaneous) muscle power relates to crank power. The reason is that there is no direct relationship between the instantaneous power delivered by a muscle and the direction/magnitude of its contribution to the pedal force (to explain this in detail goes beyond the scope of this article, but see Ref. 54). For example, this means that a muscle contributing to a large tangential pedal force may not produce any power at the muscle level, but simply “transfers” power from other muscles (see also Refs. 55 and 56). In the end, the power delivered by a muscle force is fully determined by the angular velocity of the joint(s) the muscle spans. As an example, we look again at Fig. 7*C*. In this figure, we plotted the optimal isometric muscle forces for maximal tangential force while avoiding radial force (*F*_rad_max_ = 0). We see that m. iliopsoas is substantially activated in order to compensate for the radial pedal force component of the m. soleus. However, during cycling, at this crank angle, the hip joint is extending/retro flexing, which would lead to a large instantaneous negative muscle power of m. iliopsoas. This is obviously not optimal when considering that the task is to maximize average mechanical power output over a full cycle (in the optimal control solution, this muscle is indeed not activated around this crank angle; see Fig. 6). When allowing only the use of muscles that deliver positive instantaneous muscle power, m. iliopsoas is no longer activated. Since m. iliopsoas was required to compensate for the radial pedal force component of the m. soleus, the consequence is that the m. soleus activation is reduced to almost zero (Fig. 7*D*). This is in fact a general “problem” for the muscles spanning the ankle joint during a full cycle. The ankle is close to the pedal and therefore the muscles spanning the ankle joint have a large contribution to the (radial) pedal force. Thus, when avoiding radial pedal forces, ankle spanning muscles can hardly be activated. This is the reason why m. soleus, m. gastrocnemius. and m. tibialis anterior are not able to contribute to AMPO when no radial pedal forces are allowed, leading to a 170 W reduction in AMPO (see Fig. 6).

### IE and Efficiency

One may argue (see e.g., Ref. 4) that since radial pedal forces are ineffective (not producing power) and are the consequence of active muscles that use metabolic energy, avoiding radial pedal forces may lead to more efficient cycling (i.e., less metabolic power needed for a given value of AMPO). Although this argument may sound reasonable at first glance, it is incorrect. First, the theoretical argument in introduction also holds for efficiency: if the goal is to maximize efficiency (at a given fixed value of AMPO), then adding a constraint (to avoid radial pedal forces) cannot lead to a better efficiency. Second, we can see from our analyses that avoiding radial pedal forces not only greatly diminished maximal AMPO, it also greatly increased the dissipation of power. From Fig. 6, we calculated the PDR: the ratio of negative to positive AMPO. This ratio allows us to compare the muscle power dissipation between conditions, as it is independent of the net AMPO delivered. In the unconstrained condition, PDR is about −0.2, which means that for every *W* of positive mechanical power delivered, muscles dissipate 0.2 W. PDR increases when limiting radial pedal forces and more than doubles to a value of −0.45 when completely avoiding radial pedal forces. From this increased PDR, it follows that the net amount of negative muscle power for a given amount of positive muscle power increases. This increase will result in a reduction in metabolic efficiency during sprint cycling while avoiding radial forces.

### Conclusions

Using an optimal control model of a cyclist, we showed and explained that the radial pedal forces that occur during cycling are unavoidable when maximizing average mechanical power output during a full cycle. It is true that the “ineffective” radial pedal forces do not contribute to the power delivered by the cyclist to the crank. However, the idea that avoiding these radial pedal forces will lead to more efficient pedaling is incorrect. In fact, the opposite is true: avoiding radial pedal forces causes ineffective use of muscles that not only leads to a decrease in maximal AMPO in sprint cycling, but also to a decrease in pedaling efficiency.

## APPENDIX

### Contribution of Muscle Forces to Pedal Forces

To analyze differences in outcome for different levels of constrained radial pedal forces, we calculated the (potential) contribution of each muscle to the pedal force, along the lines of the work of Zajac and coworkers (55, 56). To do so, we described our differential equations of the skeletal dynamics (see METHODS section, *Musculoskeletal Model*) as follows:

$$A\cdot {\left[{\vec{F}}_{j}\;\vec{\ddot{\varphi }}\;{\vec{F}}_{\text{con}}\;M_{\text{crank}}\;{\vec{F}}_{\text{pedal}}\right]}^{T}=\vec{b},$$

here *A* is a matrix describing the system dynamics pertaining to all unknown joint reaction forces (${\vec{F}}_{j}$), joint angular accelerations ($\vec{\ddot{\varphi }}$), constraint forces (${\vec{F}}_{\text{con}}$; forces constraining the fixed position of the hip and bottom bracket), constraint moment ($M_{\text{crank}}$; moment enforcing the constant crank angular velocity), and pedal forces (${\vec{F}}_{\text{pedal}}$). $\vec{b}$ is a vector pertaining to the gravitational and Coriolis forces and, importantly, the sum of the muscle moments around each joint. We structured our differential equations by identifying several blocks:

$$\left[\begin{array}{cc} A_{1} & A_{2}\\ A_{3} & A_{4} \end{array}\right]\cdot \left[\begin{array}{c} \vec{x}\\ {\vec{F}}_{\text{pedal}} \end{array}\right]=\left[\begin{array}{c} {\vec{b}}_{1}\\ {\vec{b}}_{2} \end{array}\right].$$

Here, $\vec{x}$ are all unknowns except ${\vec{F}}_{\text{pedal}}$. We then used some basic general linear algebra manipulations to eliminate all unknowns other than ${\vec{F}}_{\text{pedal}}$:

$${\vec{F}}_{\text{pedal}}={\left[A_{4}-A_{3}\cdot {A_{1}}^{-1}\cdot A_{2}\right]}^{-1}\cdot ({\vec{b}}_{2}-A_{3}A^{-1}\cdot {\vec{b}}_{1}).$$

This allowed us to calculate the pedal forces for any given state of the system (i.e., joint angles and angular velocities) and any net joint torque. Moreover, for any point along the cycle, we were able to use the states and joint torques obtained from our optimization and express the pedal forces as a function of net joint moments around the hip (*M*_hip_), knee (*M*_knee_), and ankle (*M*_ankle_) and a term that represents the inertial component of the pedal forces (${\vec{F}}_{\text{dyn}}$):

$${\vec{F}}_{\text{pedal}}=\left[\begin{array}{c} a_{x}\\ a_{y} \end{array}\right]M_{\text{hip}}+\left[\begin{array}{c} b_{x}\\ b_{y} \end{array}\right]M_{\text{knee}}+\left[\begin{array}{c} c_{x}\\ c_{y} \end{array}\right]M_{\text{ankle}}+{\vec{F}}_{\text{dyn}}.$$

Since the net joint moment is simply the sum of all muscle joint torques, pedal force can be expressed as a sum of eight muscle contributions:

$${\vec{F}}_{\text{pedal}}=\sum_{i=1}^{8}\left(\left[\begin{array}{c} a_{x}\\ a_{y} \end{array}\right]M_{\text{hip},i}+\left[\begin{array}{c} b_{x}\\ b_{y} \end{array}\right]M_{\text{knee},i}+\left[\begin{array}{c} c_{x}\\ c_{y} \end{array}\right]M_{\text{ankle},i}\right)+{\vec{F}}_{\text{dyn}}.$$

Here, *M*_hip_*_,i_* means the hip moment of the *i*th muscle, etc. Finally, by taking the summations apart one can calculate the contribution of any muscle to the pedal force (*F*_pedal_*_,i_*):

$${\vec{F}}_{\text{pedal},i}=\left[\begin{array}{c} a_{x}\\ a_{y} \end{array}\right]M_{\text{hip},i}+\left[\begin{array}{c} b_{x}\\ b_{y} \end{array}\right]M_{\text{knee},i}+\left[\begin{array}{c} c_{x}\\ c_{y} \end{array}\right]M_{\text{ankle},i}.$$

To give an example, for Fig. 7 we calculated the directions of the contributions of the several muscles at a crank angle of ∼55° (0° being top-dead center):

$${\vec{F}}_{\text{pedal},i}=\left[\begin{array}{c} 0.02\\ 0.03 \end{array}\right]M_{\text{hip},i}+\left[\begin{array}{c} 1.66\\ -1.43 \end{array}\right]M_{\text{knee},i}+\left[\begin{array}{c} 2.17\\ 5.95 \end{array}\right]M_{\text{ankle},i}$$

From this transfer function from joint torque to pedal force it can be appreciated that the contribution of a joint torque (and hence the muscle force producing that torque) to the pedal force greatly diminishes with increasing distance to the origin of the pedal force. In fact, for this angle, contribution of hip muscle to the pedal force is about two orders of magnitude smaller than the ankle muscles. The (normalized) direction of the force delivered by the m. soleus is [0.34 −0.94]^T^ (note that the m. soleus delivers a negative torque around the ankle joint), that of the m. vastus is [0.76 −0.65]^T^ and that of the m. iliopsoas is [0.55 0.83]^T^.

## DATA AVAILABILITY

Data will be made available upon reasonable request.

## SUPPLEMENTAL DATA

10.6084/m9.figshare.21711020 Supplemental Material: https://doi.org/10.6084/m9.figshare.21711020.

## DISCLOSURES

No conflicts of interest, financial or otherwise, are declared by the authors.

## AUTHOR CONTRIBUTIONS

D.A.K., R.M.T., E.D.H.M.R., and M.F.B. conceived and designed research; D.A.K. and R.M.T. analyzed data; D.A.K. and M.F.B. interpreted results of experiments; D.A.K. and R.M.T. prepared figures; D.A.K. drafted manuscript; D.A.K., R.M.T., E.D.H.M.R., and M.F.B. edited and revised manuscript; D.A.K., R.M.T., E.D.H.M.R., and M.F.B. approved final version of manuscript.
